# Supplementary material for: Identification of photoperiod-induced specific miRNAs in the adrenal glands of Sunite sheep (Ovis aries)
Source: Front Vet Sci. 2022 Jul 22;9:888207. doi: 10.3389/fvets.2022.888207 (PMC9354845; doi:10.3389/fvets.2022.888207)
Supplement: Supplementary file 1 [file Data_Sheet_1.ZIP › Supplementary file/miRDeep2 file.zip!/miRDeep2 file/Novel_Structure/SP42A2/Novel_180.pdf]

Provisional ID	:	NC_019480.2_38761
Score total	:	14.8
Score for star read(s)	:	3.9
Score for read counts	:	7.3
Score for mfe	:	2
Score for randfold	:	1.6
Score for cons. seed	:	
Total read count	:	26
Mature read count	:	22
Loop read count	:	0
Star read count	:	4

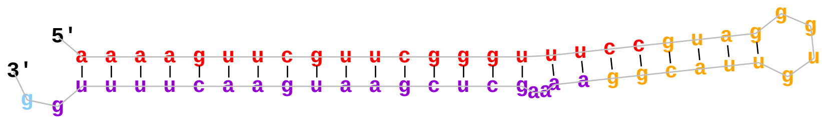

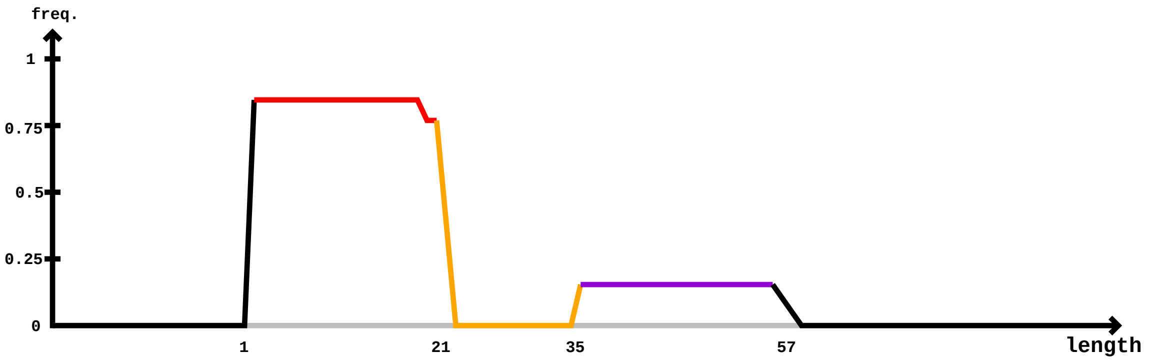

Mature Star

```
5'- cgauauacugaguuagcccaaaaguucguucggguuuccguaggguguuacggaaaagcucgaaugaacuuuugggccaacccaauacagagaacuucaggugcacaac   -3'    obs
    cgauauacugaguuagcccaaaaguucguucggguuuccguaggguguuacggaaaagcucgaaugaacuuuugggccaacccaauacagagaacuucaggugcacaac          exp
    ........((.(((.((((((((((((((((((((((((((((....))))))))..)))))))))))))))))))).))).)).........................    reads    mm    sample
    ...................aaaaguucguucggguuu........................................................................    2        0     seq
    ...................aaaaguucguucggguuuUc......................................................................    20       1     seq
    .....................................................aaaagcucAaaugaacuuuug...................................    4        1     seq
```
